# A Case of Retention of Placenta Succenturiata

**Published:** 1884-03

**Authors:** 

**Affiliations:** Berlin


### ARTICLE IV.

### A CASE OF RETENTION OF PLACENTA SUCCENTURIATA. BY DR. HAUSSMANN, of Berlin.

The following article, written by Dr. Haussmann, lecturer on gynæcology and obstetrics at the University of Berlin, throws a light upon certain conditions in the confinement of women, which to my knowledge have been hitherto entirely ignored in this country. I remember distinctly a case of this kind where the physician had deliverd the child, a placenta, and, as he thought, all of the fœtal membranes.

The woman, who had been anæmic for a number of years, died from hæmorrhage, and the autopsy proved the presence of a placenta in the uterus, with a part of the fœtal membranes. The county physician laid the blame on the doctor for having torn the placenta, thus causing fatal hæmorrhage. The latter being able to prove that he had delivered a whole placenta, denied the statement, and there was confusion on all sides.—*O. Stroinski*, M. D.

The presence of a second placenta in single births in the human female, which has to be regarded as retrogradation in the sense of Darwin, has been known for the last two centuries, and it has been observed either after spontaneous expulsion, or by hæmorrhages or by impregnation after delivery. At the time of the delivery its presence seems to have been detected only in rare cases, notwithstanding the fact that it is found in a certain number of all cases and is very well characterized by the long duration and tardiness of the delivery of the child and of the placenta. The following case, which showed characteristic symptoms at the time of the delivery, proved to me the presence of a second placenta at a time before the first placenta was delivered, and it gave me the indications for the proper action.

Mrs. X. had normal pains at the regular time, and at a quarter to 5 o'clock in the morning a living boy weighing 3,000 grams was born. About ten minutes after the birth of the child I attempted to remove the placenta by friction and pressure through the abdominal walls, the uterus contracting but little and there being considerable hæmorrhage. This attempt failed but by a second attack ten minutes later, the expression was easily performed. But in elevating the placenta and in the attempt to remove the fœtal membranes which were yet enclosed in the genital organs, I noticed a rupture in those membranes. Entering the vagina, I found a resistant tumor on the fœtal membranes, and in entering the uterus the presence of a second placenta was detected and which was adherent to the uterine walls. It was situated on the posterior wall of the uterus and adjacent to the os internum, and it was intimately connected with this wall and the fœtal membranes. In carefully separating this second placenta from the adherent wall I removed the whole from the now regularly contracting uterus, the latter being washed out with a 2 per cent. solution of carbolic acid. The patient recovered in the normal time, and the lochiæ were of the regular amount. The first placenta was normally formed, except the cotyledons, which were somewhat flat. The second placenta had a tongue-like form, and it was about 9 cm. long on its lower margin; 2 cm. on its upper surface; 5 cm. broad, and its seat on the fœtal membranes was distinctly distinguished from that of the first placenta. The method of removing the placenta by expression through the abdominal walls has now been generally accepted, but I will here call the attention of physicians to the fact that this act is sometimes made impossible by the presence of a placenta succenturiata.

